# Synthesis of Bio-Based Polyamide 6,5 via Enzymatic Polycondensation

**DOI:** 10.3390/ma19071399

**Published:** 2026-03-31

**Authors:** Yiran Xia, Shidong Sun, Qianqian Zhang, Jinlong Li

**Affiliations:** 1 Key Laboratory of Geriatric Nutrition and Health, Ministry of Education, Beijing Technology and Business University, Beijing 100048, China; xiayiran24@163.com (Y.X.); sunsd2020@126.com (S.S.); 18852862233@163.com (Q.Z.); 2 Beijing Engineering and Technology Research Center of Food Additives, Beijing Technology and Business University, Beijing 100048, China; 3 Key Laboratory of Green Manufacturing and Biosynthesis of Food Bioactive Substances, China General Chamber of Commerce, Beijing 100048, China

**Keywords:** lipase, enzymatic synthesis, polyamide 6,5

## Abstract

This work aimed to explore novel polymeric material by synthesizing polyamide 6,5 via the direct enzymatic polycondensation of dimethyl glutarate and 1,6-Diaminohexane, using the lipase Novozym 435 as a biocatalyst. While maintaining a fixed monomer feed ratio, the effects of reaction temperature, duration, and enzyme concentrations on the molecular weight and yield of the resulting polyamide 6,5 were systematically investigated. The experimental results indicated that the optimal conditions for the Novozym 435-catalyzed synthesis were a reaction time of 3 days, a temperature of 90 °C, and enzyme concentrations of 20 wt%. The establishment of this enzymatic synthesis route for polyamide 6,5 not only provides a novel methodology for polymer synthesis but also offers a new perspective for the future green materials manufacturing industry.

## 1. Introduction

Polymers serve as foundational materials in modern industry and technology. Among them, polyamides play a role in sectors such as textiles, engineering plastics, automotive manufacturing, and electronic devices due to their excellent mechanical strength, wear resistance, chemical stability, and processability [1,2,3]. The synthesis methods of polymers are crucial for developing advanced materials with broad applicability [4]. Conventional chemical synthesis routes have been widely adopted owing to their economic advantages and production capacity [5]. However, the large-scale production of traditional polyamides (e.g., PA6, PA66) heavily relies on monomers derived from fossil resources and proceeds via polycondensation processes under high temperature and pressure, which are associated with high energy consumption and a considerable environmental footprint. Their environmental impact has raised concerns, as these processes often involve the use of non-renewable resources, toxic chemicals, elevated temperatures, and significant waste generation [6,7].

In response to growing environmental challenges, the enzymatic synthesis of polymers has emerged as a promising alternative to conventional chemical methods. Against the backdrop of global efforts toward “dual-carbon” goals and the transition to green manufacturing, developing new environmentally benign, mild, and sustainable routes for polymer synthesis has become a key frontier in chemistry, materials science, and engineering. Bio-based polymers are derived from monomers obtained via the chemical or biological breakdown of renewable biomass resources, such as wheat, corn, sorghum, and straw, followed by polymerization [8,9,10,11]. Bio-based polyamides can partially replace their fossil-based counterparts, reducing the consumption of non-renewable resources [12,13,14].

The lipase-catalyzed synthesis of polyamides can significantly lower energy consumption and equipment demands, making the production process more environmentally friendly, sustainable, and energy-efficient. Enzyme catalysis offers a highly potential green pathway for polymer synthesis [15,16,17,18,19]. Compared with traditional metal catalysts, enzymes exhibit notable advantages such as high efficiency, superior selectivity, mild reaction conditions (often at ambient temperature and pressure), good biocompatibility, and biodegradability [15,20,21]. Lipases are among the most widely used enzymes in organic synthesis [22,23]. As biocatalysts, they can catalyze a series of hydrolysis or synthesis reactions in both aqueous and non-aqueous media [24,25]. Lipases are stable and robust enzymes that remain active toward various non-natural substrates. Among numerous enzymes, lipases have become one of the most favored catalysts for enzyme-catalyzed polycondensation research due to their ability to catalyze amide bond formation and their good stability in non-aqueous media. Novozym 435 (immobilized Candida antarctica lipase B) is one of the most widely used commercial catalysts, as the lipase has the ability to catalyze amide bond formation and has a good stability in non-aqueous media [26,27,28,29,30].

Enzymatic crosslinking and polymerization strategies have garnered burgeoning interest in the functional materials field in recent years: they not only embody the core demands for green advanced material development, but also enable the precise, selective regulation and tunable construction of material architectures in bio-based and advanced polymer systems, emerging as a powerful and versatile tool for designing high-performance functional materials, with a wealth of recent high-impact studies continuously demonstrating their great application potential in this research frontier [31,32,33].

In this study, a systematic investigation was designed and conducted for immobilized lipase (Novozym 435) for the polycondensation of dimethyl glutarate and 1,6-Diaminohexane, aiming to efficiently and sustainably synthesize bio-based polyamide 6,5. Dimethyl glutarate was synthesized via a green esterification process. This builds upon the work of Chu et al. [34], who developed an efficient, plasmid-free, antibiotic-free, and inducer-free route for bio-glutaric acid production from glucose using an engineered strain. Bio-based dimethyl glutarate is ultimately produced via the reaction of glutaric acid with bio-based methanol [35]. Using Novozym 435 as biocatalyst, the effects of sythesized temperature, time, and enzyme concentrations were examined and optimized. This bio-based polyamide 6,5 was characterized through several methods. This study presents a more sustainable and eco-friendly process for synthesizing polyamide 6,5 via enzymatic catalysis. The resulting polyamide 6,5 exhibits a lower melting point compared to conventional polyamides, endowing it with enhanced spinnability. This property warrants further investigation for its potential in fiber production and related applications.

This work is the first report of CALB-catalyzed polycondensation in an inhomogeneous dispersion system for polyamide 6,5 synthesis. Regarding the synthesis of polyamide 6,5 via this route, it represents a significant advance compared with prior studies: the existing polyamide 6,5 synthesis mostly relies on harsh reaction conditions, while this work optimizes the enzymatic synthesis route to improve molecular weight, filling the gap in CALB-catalyzed polyamide 6,5 synthesis in inhomogeneous systems.

## 2. Materials and Methods

### 2.1. Materials

Novozym 435 [N435, Candida antartica lipase B (CALB) immobilized on acrylic resin, ≥5000 U g^−1^], 1,6-hexanediamine (1,6-HDA, 98%), dimethyl glutarate (DMG, 98%), toluene(C_7_H_8_, 99.8%), formic acid (C_2_H_2_O), tetra hydrofuran (THF, C_4_H_8_O), hex afluoroisopropanol (HFIP) (C_3_H_2_F_6_O), trifluoroacetic acid-d (TFA-d), potassium bromide (KBr) (FTIR grade), and the ZSM-5 molecular sieve (SiO_2_/Al_2_O_3_ ≈ 25–30) were provided by Macklin (Shanghai, China).

### 2.2. Enzymatic Synthesis of Polyamide 6,5

**Scheme 1:**
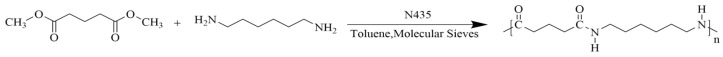
Lipase Novozym 435-catalyzed synthesis of bio-based polyamide 6,5.

Dimethyl glutarate (0.05 mol), 1,6-Diaminohexane (0.05 mol), toluene (5 mL), pre-dried lipase, and molecular sieves were placed in a 50 mL round-bottom flask. The reaction mixture was placed under reduced pressure (20–40 mm Hg) with magnetic stirring set at 120 rpm. The polymerization was allowed to proceed for a specified duration. Upon completion, toluene was removed via rotary evaporation. The crude product was then thoroughly washed with formic acid to ensure the complete dissolution of polyamide. The lipase and molecular sieves were removed through filtration. The filtrate was concentrated to approximately 2–5 mL using rotary evaporation. The concentrated solution was poured into excess THF and stored at −20 °C for 24 h to precipitate the polymer. The precipitate was collected through centrifugation, followed by vacuum drying at 40 °C for 3 days, yielding the final polyamide 6,5 product. The reaction scheme for the lipase Novozym 435-catalyzed synthesis of polyamide 6,5 is presented in Scheme 1.

### 2.3. Analytical Techniques

Fourier transform infrared (FTIR) spectroscopy (Thermo Nicolet iS5, Thermo Fisher Scientific) was employed to characterize the dried polyamide 6,5 product. Spectra were acquired with 32 scans at a resolution of 4 cm^−1^. Nuclear magnetic resonance (NMR) spectroscopy was used to characterize the chemical structure of the enzymatically synthesized polyamide 6,5. The sample was dissolved in trifluoroacetic acid-d (TFA-d) for analysis. ^1^H spectra were acquired at room temperature on a Bruker AVANCE III HD 500 MHz spectrometer. The ^1^H NMR measurements were conducted at a resonance frequency of 500 MHz. Chemical shifts are reported in parts per million (ppm). Elemental analysis (EA) of the polyamide 6,5 sample was performed using an Elementar Vario Micro cube analyzer to determine the contents of carbon (C), hydrogen (H), oxygen (O), and nitrogen (N). The measured values were compared with the theoretical values, and the elemental deviation rate was calculated as follows: (Found Value − Theoretical Value)/Theoretical Value ×100%. MALDI-TOF MS analysis was performed on a Bruker Ultraflextreme spectrometer operating in reflector mode, with an accumulation of 100 laser shots per spectrum. 2,5-Dihydroxybenzoic acid (DHB) was used as a matrix, hexafluoroisopropanol (HFIP) was used as a solvent, and sodium trifluoroacetate was added for cationization. Gel permeation chromatography (GPC) was employed to determine the number-average molecular weight (M_n_), weight-average molecular weight (M_w_), and polydispersity index (PDI) of the synthesized polyamide 6,5. The analysis was performed using an Agilent PL-GPC 50 system. Polyamide 6,5 was dissolved in hexafluoroisopropanol (HFIP), and redistilled HFIP was used as the mobile phase at a flow rate of 1 mL min^−1^ with a column temperature of 40 °C. Differential scanning calorimetry (DSC) analysis was performed using the NETZSCH 3500 calorimeter. Approximately 10 mg of the sample was sealed in an aluminum pan and subjected to a heat–cool–heat cycle from 25 to 300 °C under nitrogen atmosphere with a flow rate of 40 mL min^−1^. Both heating and cooling rates were set at 10 °C min^−1^. Thermogravimetric analysis (TGA) was performed on the synthesized polyamide using a HITACHI STA200 analyzer under nitrogen atmosphere, with a heating rate of 10 °C min^−1^ from 30 to 600 °C. X-ray diffraction (XRD) analysis was performed using a Bruker D8 Advance diffractometer. Diffraction voltage was set to 40 kV, current to 40 mA, slit width to 1.0 mm, scan rate to 10°/min, and step size to 0.02°, with a scan range from 10° to 80°.

## 3. Results and Discussion

### 3.1. Qualitative Identification of Bio-Based Polyamide 6,5

**Figure 1:**
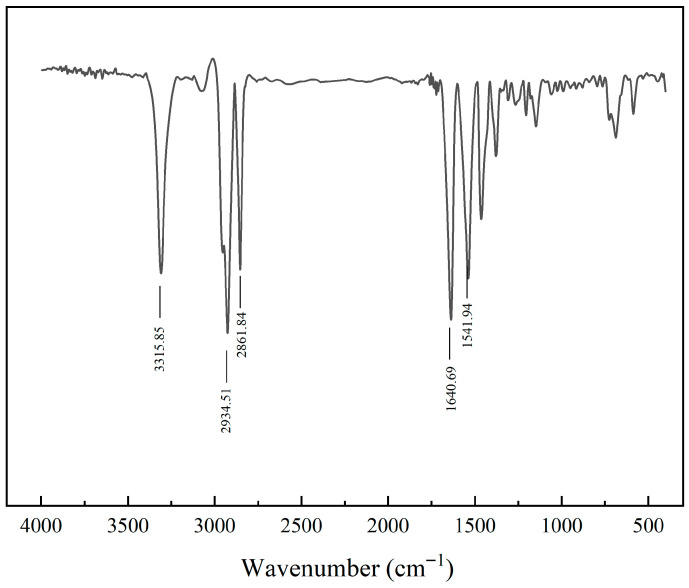
FTIR spectra of polyamide 6,5.

Figure 1 displays the FTIR spectrum of bio-based polyamide 6,5 synthesized through enzymatic methods, achieving the highest relative molecular mass. The FTIR analysis indicates that the bio-based polyamide 6,5 produced with lipase Novozym 435 exhibits distinct absorption bands characteristic of amide groups, thereby confirming the successful enzymatic synthesis of polyamide 6,5. The prominent absorption peaks at 3309.41 cm^−1^ (N-H stretching), 1636.61 cm^−1^ (C=O stretching), 1539.42 cm^−1^ (N-H bending), and 1277 cm^−1^ (C-N stretching) are all associated with the amide group. Additionally, the spectral peaks at 2924.28 cm^−1^ (CH_2_ asymmetric stretching) and 2854.32 cm^−1^ (CH_2_ symmetric stretching) correspond to C-H stretching vibrations of saturated hydrocarbon bonds. The presence of characteristic peaks from the amide group suggests that dimethyl glutarate underwent condensation polymerization with 1,6-Diaminohexane to form amide bonds, resulting in the production of nylon 6,5. This finding confirms the overall success of lipase-catalyzed polyamide synthesis.

**Figure 2:**
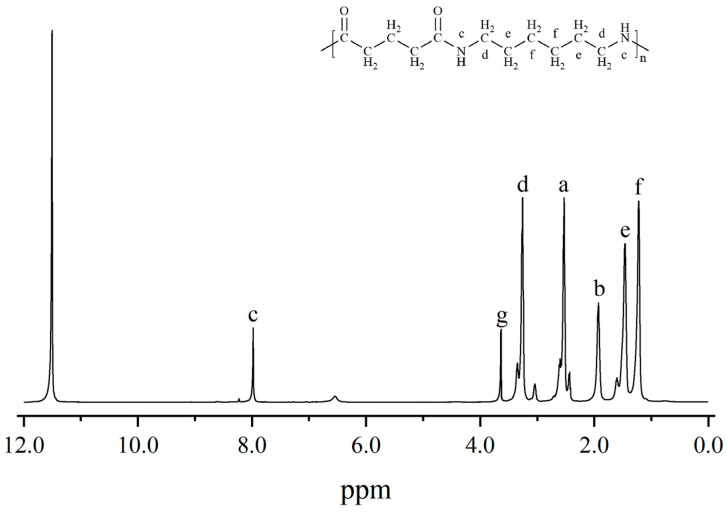
^1^H NMR spectrum of polyamide 6,5. Lowercase letters (a–g) correspond to the peak labels shown in the table.

The ^1^H NMR spectrum of the synthesized polyamide 6,5 recorded in deuterated trifluoroacetic acid (TFA-d) is shown in Figure 2. The characteristic proton signals were assigned as follows (^1^H NMR, 500 MHz, TFA-d, δ): 7.98 (s, 1H), 3.63 (s, 3H), 3.26 (s, 4H), 2.53 (t, 4H), 1.92 (m, 2H), 1.47 (m, 4H), and 1.22 (m, 4H). The signal at 7.98 ppm is attributed to the amide proton (–NH–CO–). The resonances at 2.53 ppm (–CH_2_–CONH–) and 1.92 ppm (–CH_2_–CH_2_–CONH–) correspond to the methylene protons in the glutarate unit, while the signals at 3.26 ppm (–CONH–CH_2_–), 1.47 ppm (–CONH–CH_2_–CH_2_–), and 1.22 ppm (–CONH–CH_2_–CH_2_–CH_2_–) arise from the methylene protons in the diamine unit. The signal at 3.63 ppm (CH_3_–CONH–) corresponds to the protons of the terminal methoxy ester groups. Combined with the FTIR results, the ^1^H NMR data confirm the successful synthesis of polyamide 6,5.

The EA results for the enzymatically synthesized polyamide 6,5 are presented in Table 1. The polyamide macromolecule consists of four elements: C, H, O, and N. Theoretical mass percentages for each element in the structural unit were calculated based on the molecular formula of nylon 6,5. The experimental mass percentages of C, H, O, and N in the synthesized material were determined using an elemental analyzer and compared with the theoretical values to calculate deviation rates. As shown by the data in Table 1, the deviations between experimental and theoretical values are minor, with all deviation rates below 2%. This close agreement confirms that the elemental composition of the synthesized product aligns well with the expected composition of the polyamide 6,5 structural unit.

**Figure 3:**
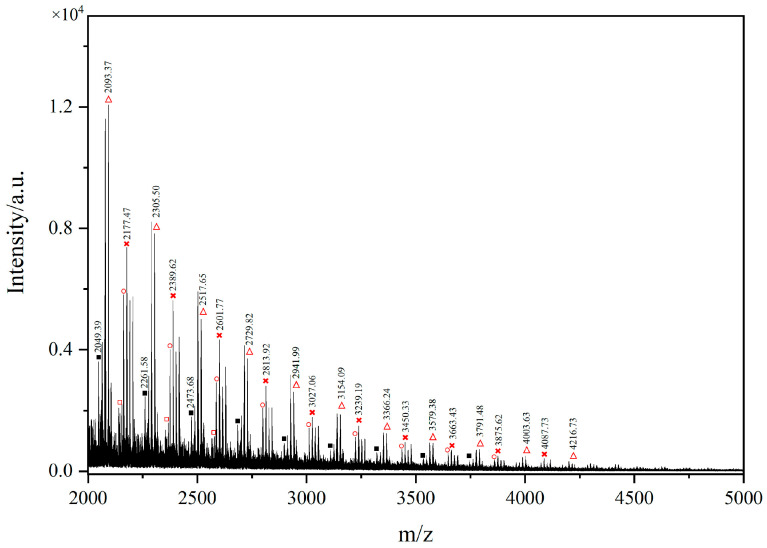
MALDI-TOF MS analysis of polyamide 6,5.

MALDI-TOF MS analysis was performed on the polyamide 6,5 sample with a relatively low M_n_. As shown in Figure 3 and Table 2, the MALDI-TOF mass spectrum extended to *m*/*z* 4500 and displayed an ion distribution corresponding to sodium adducts (M + Na^+^). The peak-to-peak mass increment was determined to be 212.3 Da, which is in perfect agreement with the mass difference between consecutive oligomeric species. The spectrum also revealed that the oligoamide series was terminated by four distinct identifiable end-group configurations: amine/ester, amine/acid, amine/amine, and ester/ester. A low proportion of cyclic oligoamides was detected in the MALDI-TOF mass spectrum.

A comparison of the polyamide 6,5 synthesized in this study via enzymatic catalysis with that produced through conventional chemical methods in previous research revealed consistent structural characteristics, confirming the successful synthesis of polyamide 6,5 using lipase [36].

### 3.2. Synthesis Effectiveness of Bio-Based Polyamide 6,5

The lipase-catalyzed synthesis of polyamide is a reversible process where the equilibrium between polycondensation and hydrolysis directly dictates the product’s molecular weight and yield [37,38]. To enhance the polymerization efficiency of polyamide 6,5, the core strategy employed here is to shift the reaction equilibrium toward polycondensation. This objective was achieved by enhancing the forward reaction rate and removing water vapor from the vessel under high-temperature conditions: the reaction was conducted under a vacuum with the addition of molecular sieves to adsorb moisture [39,40]. The presence of water favors the hydrolytic equilibrium, leading to the decomposition of both substrates and products, which consequently lowers the polymerization yield and molecular weight. Therefore, utilizing a non-aqueous reaction medium (e.g., organic solvent or ionic liquid) effectively limits water concentration, suppresses hydrolysis, and establishes polycondensation as the dominant pathway. Within non-aqueous media, solvent polarity critically influences enzyme activity and reaction equilibrium. As reported by Kumar et al. [27], the solvent’s logP serves as the key parameter: solvents with a lower logP (higher polarity) hinder polymerization, typically yielding lower molecular weights, whereas those with a higher logP (greater hydrophobicity) promote enzymatic polycondensation [41,42]. Based on a comprehensive evaluation of the solvent boiling point, safety, and logP, this study selected toluene as the reaction medium. This choice aims to minimize water interference while providing an appropriate microenvironment for the enzyme, thereby optimizing the synthesis of polyamide 6,5. The reaction scheme for the lipase Novozym 435-catalyzed synthesis of polyamide 6,5 is presented in Scheme 1.

Polyamide 6,5 was synthesized from dimethyl glutarate and 1,6-Diaminohexane via lipase Novozym 435 catalysis. The effects of reaction temperature, time, and enzyme concenration on polymerization were evaluated based on product molecular weight and monomer yield. The molecular weights and yields of polyamide 6,5 prepared under different conditions using Novozym435 are summarized in Table 3.

The results indicate that, within the investigated temperature range, the lipase maintained a relatively high activity at elevated temperatures. Increasing the temperature effectively accelerated the catalytic reaction, enhanced the conversion of substrates, and significantly promoted the chain growth of polyamide 6,5, thereby yielding a higher molecular weight. Under the conditions of 72 h reaction time and 20 wt% enzyme concentrations, an M_n_ of 6900 g mol^−1^ with a yield of 63% was achieved at 80 °C, while, at 90 °C, the M_n_ increased to 8600 g mol^−1^ with a yield of 72%. When the reaction temperature reached 100 °C and above, the chain length of the polyamide approached a critical level, and further increases in temperature resulted in only minor changes in M_n_ and yield. Within the 80–90 °C range, the well-preserved enzyme activity led to a notable increase in both the M_n_ and yield. At temperatures of 100 °C and higher, the activity of the lipase diminished, and physical changes in the reaction system, such as increased diffusion viscosity, contributed to the observed plateau in M_n_ and yield.

At a reaction temperature of 90 °C and enzyme concentration of 20 wt%, both the M_n_ and the yield of polyamide 6,5 increased with extended reaction times. The present polycondensation was conducted in an inhomogeneous dispersion system, which is a key factor leading to the difficulty in reaction control. During the polymerization process, the polyamide products were observed to precipitate out at 24 h of reaction. At 24 h, the polymer exhibited an M_n_ of 4300 g mol^−1^ with a corresponding yield of 39%. This precipitation behavior was closely related to the solubility of the polyamide in the reaction system and the gradual increase in molecular weight during polymerization. To clarify whether chain propagation could proceed after precipitation, further observations and analyses were performed: it was found that the polymerization reaction still continued to a certain extent after precipitation because the enzyme adsorbed on the surface of the precipitated products could still catalyze the reaction between residual monomers or terminal groups of the precipitated polymers. A significant enhancement was observed between 24 h and 72 h, where the M_n_ rose from 4300 to 8600 g mol^−1^ and the yield increased by 33%. However, upon reaching 96 h, a further extension of the reaction time did not lead to a notable improvement in either the M_n_ or yield, indicating that the system approached a plateau.

At fixed reaction temperature of 90 °C and reaction time of 72 h, both the M_n_ and yield of polyamide 6,5 were relatively low at lower lipase concentrations. The results indicated that the M_n_ and yield increased with increasing enzyme concentrations. A marked increase was observed when the enzyme concentration reached 20 wt%. When further increased to 25 wt%, the enzymatic synthesis achieved its highest values, with an M_n_ of 8900 g mol^−1^ and yield of 75%. However, the incremental improvement from 20 to 25 wt% was marginal, as the product synthesis had already reached a high level at a 20 wt% enzyme concentration. Therefore, considering overall efficiency, the optimal enzyme concentration for the lipase-catalyzed synthesis of polyamide 6,5 was determined to be 20 wt%. The lipase-catalyzed synthesis of polyamide constitutes an equilibrium system, where reverse reaction proceeds concurrently with polymerization. In the initial stages, optimizing reaction conditions favors the forward reaction more significantly than the reverse, shifting the equilibrium toward polymer formation. However, once conditions reach a certain level, factors such as the accumulation of product, changes in the reaction system’s physical properties (e.g., increased viscosity), and potential alterations in lipase activity begin to influence the equilibrium. These factors can inhibit further forward synthesis, increase the contribution of the reverse reaction, and ultimately lead to a plateau or even decline in the M_n_ and yield. Based on the findings above, in the N435-catalyzed polycondensation of dimethyl glutarate and 1,6-Diaminohexane, temperature, time, and enzyme concentration all significantly influenced the M_n_ and yield of polyamide 6,5. Appropriate conditions for synthesis were determined to be a reaction time of 3 days, a temperature of 90 °C, and an enzyme loading of 20 wt%.

### 3.3. Characterization of Bio-Based Polyamide 6,5

**Figure 4:**
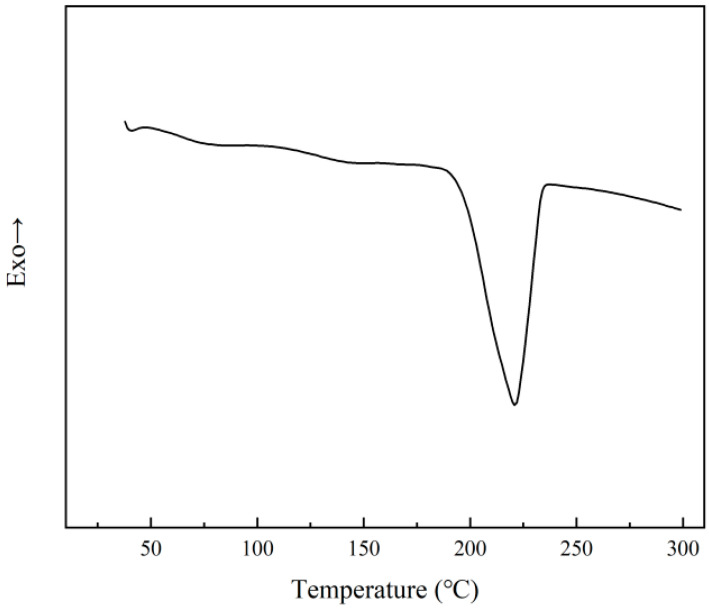
DSC second heating curve of bio-based polyamide 6,5.

**Figure 5:**
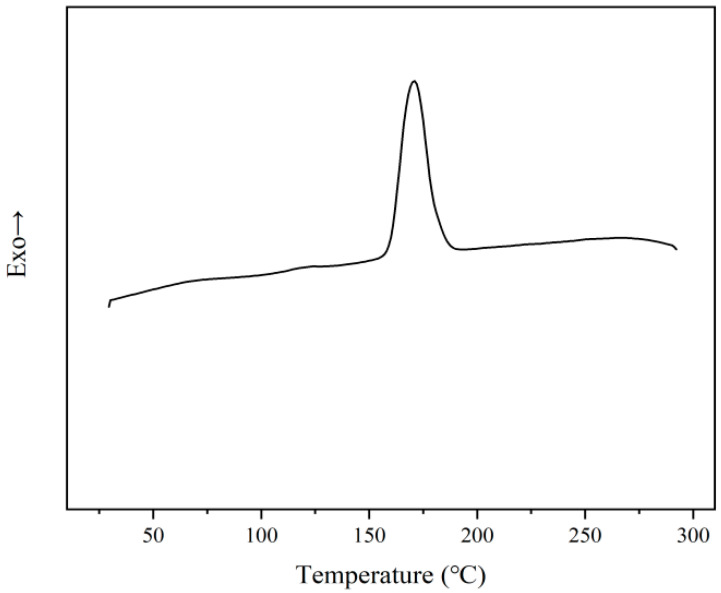
DSC cooling curve of bio-based polyamide 6,5.

DSC was employed to analyze the thermal behavior of the synthesized polyamide 6,5. The cooling and second heating profiles were obtained from the heating–cooling–heating cycle, as presented in Figure 4 and Figure 5, respectively. From these curves, the melting point (T_m_), crystallization temperature (T_c_), and enthalpy of fusion of the polymer were determined. Based on the DSC curve and analysis data of polyamide 6,5, its melting point was determined to be 221 °C, with a crystallization temperature of 170 °C. As shown in Figure 5, the values of T_m,on_, T_m,end_, and ∆H_m_ were 196 °C, 223 °C, and 62.06 J g^−1^, respectively. Based on Figure 4, the values of T_c,on_, T_c,end_, and ∆H_c_ are 160 °C, 181 °C, and 61.09 J g^−1^.

In terms of thermal properties, the polyamide 6,5 synthesized via lipase catalysis exhibited lower T_m_ and T_c_ values than those reported for chemically synthesized polyamide 6,5 in previous studies. In a study by Navarro et al. [36]., polyamide 6,5 displayed a T_c_ of 206 °C and T_m_ values of 224 °C and 239 °C. In contrast, the enzymatically synthesized polyamide 6,5 in this work showed a T_c_ of 170 °C and T_m_ of 221 °C. The moderately reduced melting point and significantly lower crystallization temperature suggest an enhanced processability for subsequent manufacturing. Furthermore, while conventionally synthesized polyamide 6,5 presented a double melting peak—likely attributable to complex crystalline morphology or polymorphic transitions—the polymer obtained in this study exhibited a single melting endotherm at 221 °C. This indicates more uniform and well-defined crystal structure, which is advantageous for achieving final products with improved performance stability.

**Figure 6:**
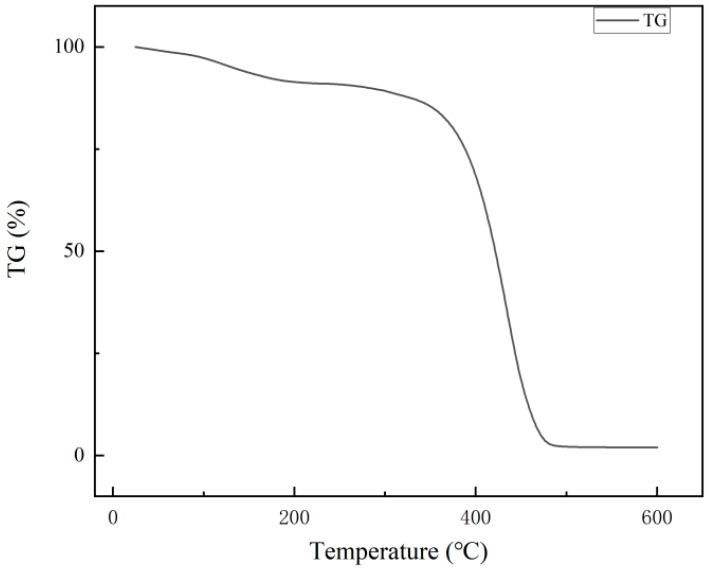
TGA curve of bio-based polyamide 6,5.

TGA was employed to characterize the thermal stability of the synthesized polyamide 6,5. As shown in Figure 6, the TGA curve displayed a single, smooth mass-loss step corresponding to a one-stage decomposition process. The maximum decomposition temperature (T_max_) was determined to be 434 °C. Below 300 °C, prior to the onset of major thermal decomposition, the TGA curve exhibits a minor and broad mass loss, which is typically not attributable to a main-chain cleavage of the polymer. Polyamide 6,5 was synthesized via lipase catalysis, a relatively mild polymerization method. Following polymerization, residual linear or cyclic oligomers, as well as solvents introduced during the reaction or post-treatment, may remain in the product. These low-molecular-weight species possess a thermal stability significantly lower than that of high-molecular-weight polyamide 6,5. During TGA heating, they gradually volatilize at temperatures well below the melting point and decomposition temperature of the nylon, resulting in a gentle, broad mass-loss plateau in the TGA curve. Due to their low content and wide volatilization temperature range, this mass-loss step manifests as “minor and broad”.

**Figure 7:**
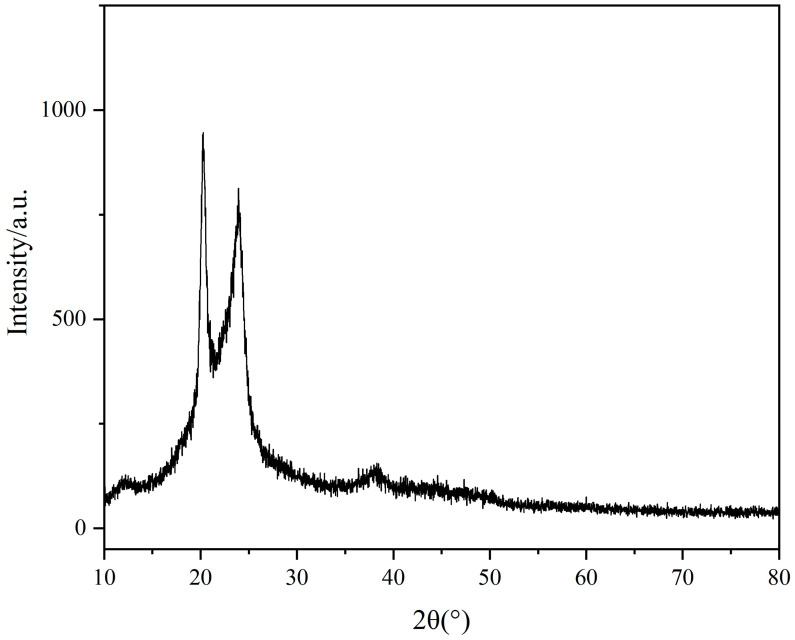
XRD diffractogram of polyamide 6,5.

Figure 7 presents the XRD pattern of polyamide 6,5, which exhibits sharp diffraction peaks accompanied by distinct amorphous diffuse scattering background. The characteristic sharp peaks at 20.27° (200 plane) and 23.93° (002 plane) clearly indicate that the sample is predominantly composed of α-form crystals, with no shoulder peaks near 21° or 23° corresponding to γ-form, confirming phase purity without α/γ mixed crystallization. The α-form represents the stable crystal phase of polyamide 6,5, and its intrinsic thermal behavior is characterized by a single melting endotherm, consistent with the DSC result for enzymatically synthesized polyamide 6,5, which presented a single melting peak. Combined with the sharp peak shape in the XRD pattern, it can be further concluded that the crystalline regions possess a high degree of order and uniform crystallite size, without significant crystalline defects.

The enzymatically synthesized polyamide 6,5 exhibits excellent thermal resistance. In current industrial production, the typical spinning temperature for common polyamides ranges from approximately 270 to 290 °C. Within this temperature range, polyamide 6,5 maintains a strong stability, demonstrating its suitability for melt-spinning processes.

## 4. Conclusions

In this study, polyamide 6,5 was synthesized from dimethyl glutarate and 1,6-Diaminohexane using lipase Novozym 435 as a biocatalyst and toluene as a solvent under a vacuum. This research holds significance because bio-based polyamides can partially replace fossil-based counterparts. The enzymatic route substantially reduces energy consumption and equipment demands, leading to production processes that are more environmentally friendly, energy-efficient, and sustainable. Following an investigation into lipase-catalyzed amidation, the optimal conditions were determined to be a reaction time of 3 days, a temperature of 90 °C, and enzyme concentrations of 20 wt%. Under these conditions, the product achieved an Mn of 8600 g mol^−1^ with a yield of 72%. Comprehensive structural and thermal characterization confirmed the successful synthesis of polyamide 6,5 and provided key parameters regarding its thermal stability, crystallization, and melting behavior. The enzymatically synthesized polyamide 6,5 exhibits excellent thermal properties, making it well suited for industrial processes like fiber spinning. These data furnish a solid experimental foundation for assessing the future application potential of polyamide 6,5.

## Figures and Tables

**Table 1:** Elemental analysis data of the sythesized polyamide 6,5.

Element	Found (%)	Theoretical (%)	Deviation (%)
C	57.04	57.37	0.58
	56.98		0.68
	57.13		0.42
H	9.53	9.63	1.06
	9.73		1.02
	9.62		0.10
O	21.05	20.84	1.02
	21.23		1.88
	21.05		1.01
N	12.38	12.16	1.81
	12.06		0.82
	12.20		0.33

**Table 2:** Polyamide 6,5 microstructures with different end groups.

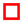	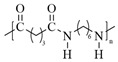	Cycles, RM = 0 amu
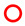	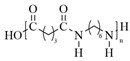	Acid/amine, RM = 18 amu
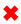	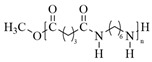	Ester/amine, RM = 32 amu
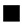	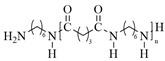	Diamine, RM = 116.2 amu
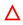	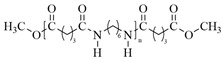	Diester, RM = 160.2 amu

RM, remaining mass.

**Table 3:** The GPC data of the synthesized polyamide 6,5.

Temperature (°C)	Time (h)	Enzyme Concentrations (wt%)	M_n_	M_w_	PDI	Yield (%)
80	72	20	6900	11,600	1.68	63
90	72	20	8600	14,100	1.64	72
100	72	20	8800	15,500	1.76	80
110	72	20	8800	15,600	1.77	83
90	24	20	4300	7400	1.72	39
90	48	20	6800	11,500	1.69	58
90	72	20	8600	14,100	1.64	72
90	96	20	8400	14,800	1.76	75
90	72	10	5800	9200	1.59	55
90	72	15	6800	11,400	1.68	62
90	72	20	8600	14,100	1.64	72
90	72	25	8900	15,500	1.74	75

## Data Availability

The original contributions presented in this study are included in the article. Further inquiries can be directed to the corresponding author.
